# Oxygen-Sensitive K^+^ Channels Modulate Human Chorionic Gonadotropin Secretion from Human Placental Trophoblast

**DOI:** 10.1371/journal.pone.0149021

**Published:** 2016-02-10

**Authors:** Paula Díaz, Colin P. Sibley, Susan L. Greenwood

**Affiliations:** 1 Maternal and Fetal Health Research Centre, Institute of Human Development, The University of Manchester, Manchester Academic Health Science Centre, Manchester, United Kingdom; 2 St. Mary's Hospital, Central Manchester University Hospitals NHS Foundation Trust, Manchester, United Kingdom; University of Oxford, UNITED KINGDOM

## Abstract

Human chorionic gonadotropin (hCG) is a key autocrine/paracrine regulator of placental syncytiotrophoblast, the transport epithelium of the human placenta. Syncytiotrophoblast hCG secretion is modulated by the partial pressure of oxygen (*p*O_2_), reactive oxygen species (ROS) and potassium (K^+^) channels. Here we test the hypothesis that K^+^ channels mediate the effects of *p*O_2_ and ROS on hCG secretion. Placental villous explants from normal term pregnancies were cultured for 6 days at 6% (normoxia), 21% (hyperoxia) or 1% (hypoxia) *p*O_2_. On days 3–5, explants were treated with 5mM 4-aminopyridine (4-AP) or tetraethylammonium (TEA), blockers of *p*O_2_-sensitive voltage-gated K^+^ (K_V_) channels, or ROS (10–1000μM H_2_O_2_). hCG secretion and lactate dehydrogenase (LDH) release, a marker of necrosis, were determined daily. At day 6, hCG and LDH were measured in tissue lysate and ^86^Rb (K^+^) efflux assessed to estimate syncytiotrophoblast K^+^ permeability. hCG secretion and ^86^Rb efflux were significantly greater in explants maintained in 21% *p*O_2_ than normoxia. 4-AP/TEA inhibited hCG secretion to a greater extent at 21% than 6% and 1% *p*O_2_, and reduced ^86^Rb efflux at 21% but not 6% *p*O_2_. LDH release and tissue LDH/hCG were similar in 6%, 21% and 1% *p*O_2_ and unaffected by 4-AP/TEA. H_2_O_2_ stimulated ^86^Rb efflux and hCG secretion at normoxia but decreased ^86^Rb efflux, without affecting hCG secretion, at 21% *p*O_2_. 4-AP/TEA-sensitive K^+^ channels participate in *p*O_2_-sensitive hCG secretion from syncytiotrophoblast. ROS effects on both hCG secretion and ^86^Rb efflux are *p*O_2_-dependent but causal links between the two remain to be established.

## Introduction

The endocrine and nutrient transport functions of the human placenta depend on appropriate maintenance of syncytiotrophoblast, a highly specialised multinucleate epithelial cell. Syncytiotrophoblast has a short life span and is renewed during pregnancy by cellular turnover. Proliferative mononucleate cytotrophoblasts exit the cell cycle, differentiate and fuse into the overlying syncytiotrophoblast and then aged syncytial nuclei are removed, possibly by apoptosis and autophagy, to complete turnover [1, 2]. In normal pregnancy these processes are highly coordinated but in pregnancies complicated by pre-eclampsia [3, 4], fetal growth restriction [4] and maternal obesity [5], an imbalance in cell turnover dysregulates syncytiotrophoblast renewal which compromises function and contributes to maternal and fetal mortality, and morbidity associated with these pregnancy complications.

Cell turnover to renew syncytiotrophoblast is maintained by several hormones including human chorionic gonadotrophin (hCG). hCG is an autocrine/paracrine regulator of syncytiotrophoblast renewal, acting via the G-protein coupled luteinizing hormone/hCG-receptor to elevate cAMP/protein kinase A and promote cytotrophoblast differentiation [6], gap junction communication and cellular fusion to form multinucleated syncytia [7]. hCG is synthesized and secreted by terminally differentiated syncytiotrophoblast and promotes continued trophoblast renewal by positive feedback. Thus appropriate regulation of hCG synthesis and secretion is essential for maintenance of syncytiotrophoblast and successful pregnancy.

Syncytiotrophoblast hCG secretion is modulated *in vitro* by oxygen tension (*p*O_2_) and reactive oxygen species (ROS). Lowering *p*O_2_ inhibited hCG secretion by villous explants [8] and by primary cultures of cytotrophoblasts [9] from normal term placentas. Hydrogen peroxide (H_2_O_2_) treatment of cytotrophoblasts, to generate oxidative stress, inhibited hCG secretion at high (>50μM) but markedly stimulated secretion at lower (1–50μM) concentrations [10]. Modulation of hCG secretion by these factors is likely to be of pathophysiological significance in pre-eclampsia and fetal growth restriction as altered placental *p*O_2_ and increased placental oxidative stress are associated with these conditions [11–13]. Indeed, increased levels of markers of oxidative stress are found in placental tissue from women with pre-eclampsia [14, 15] as well as elevated serum levels of H_2_O_2_ compared to normal pregnancies [16]. In addition to modulating hCG secretion, altered *p*O_2_ and elevated ROS also dysregulate syncytiotrophoblast turnover *in vitro* [8, 17, 18], but the underlying mechanism/s are currently unexplored.

hCG secretion by term placental trophoblast involves constitutive release [19] and Ca^2+^-dependent exocytosis [20]. Accordingly, the regulated component of hCG secretion is modulated by factors that influence intracellular Ca^2+^, including ion channels. We have previously shown that pharmacological blockade of Ca^2+^ entry channels [21] and voltage-gated K^+^ (K_V_) channels, inhibit hCG secretion from placental villous explants and isolated cytotrophoblasts [22]. Blocking K_V_ channels also inhibits trophoblast fusion to form multinucleate syncytia [22] suggesting that activity of these channels is required both for hCG secretion and syncytiotrophoblast renewal.

The K_V_ channel family comprises 11 members [23], and the expression/activity of some K_V_ channel subunits is acutely and chronically modulated by *p*O_2_ [24, 25]. *p*O_2_-sensitive K_V_ channels close in response to lowered *p*O_2_, raising the possibility that the reduction in hCG secretion from syncytiotrophoblast under hypoxic conditions is a result of blocking K_V_ channels. Furthermore, long term exposure to oxidative stress (ROS) alters K^+^ channel expression/activity and acute exposure has direct effects on K^+^ channel proteins to alter their activity [26, 27]. The effects of H_2_O_2_ are diverse and depend on tissue type; H_2_O_2_ has been reported to both close [28] and open [29, 30] K_V_ channels. As K_V_ channels are modulated by ROS in non-placental tissue, it is plausible that ROS regulate syncytiotrophoblast hCG secretion through effects on K_V_ channels.

Here we test the hypothesis that *p*O_2_ and/or ROS regulate hCG secretion through an effect on K^+^ channels. Using placental villous tissue from normal term pregnancy we compared the effect of K_V_ channel blockers on hCG secretion and ^86^Rb efflux (a marker of K^+^ permeation through ion channels) from villous explants maintained at placental normoxia (6% *p*O_2_), with extreme hypoxia (1% *p*O_2_) and hyperoxia (21% *p*O_2_). We also investigated the effect of H_2_O_2_, used to generate ROS, on hCG secretion and ^86^Rb efflux at the three different *p*O_2_.

## Materials and Methods

### Materials

Unless otherwise stated, all chemicals were from Sigma-Aldrich (Poole, UK).

### Ethics Statement

Human placentas used in this study were obtained from St. Mary’s Hospital Maternity Unit (Manchester, UK) following written informed consent as approved by the Local Research Ethics Committee (North West—Haydock Research Ethics Committee (Ref: 08/H1010/55), Central Manchester University Hospitals NHS Foundation Trust). Normal term placentas (37–42 weeks gestation) were obtained from uncomplicated pregnancies following vaginal delivery or Caesarean section. 3–14 placentas were collected depending on the type of experiment. The investigation conforms to the principles outlined in the Declaration of Helsinki.

### Placental villous explant culture

Term placental villous tissue maintained in explant culture is a well characterised model [31] which has been used extensively to study the chronic effects of regulators on syncytiotrophoblast biology [8, 17, 18] and the method for culture of villous explants has been published previously [22, 31]. Briefly, chorionic villous sections (1.5cm^3^) were sampled, further dissected into explants (3–5mm^3^) and cultured at 37°C in explant culture medium (10% CMRL-1066, 100μg/ml streptomycin sulphate, 100IU/ml penicillin-G, 0.1μg/ml hydrocortisone, 0.1μg/ml retinol acetate, 0.1μg/ml insulin, 5% fetal calf serum, pH 7.2). Explants were placed onto Netwell permeable supports (70μM mesh; Corning Costar, Loughborough, UK) at the air/liquid interface and cultured in humidified incubators at 6% *p*O_2_ (with 5% CO_2_/balance N_2_; normoxic for term placenta, 40–50mmHg; assuming 1atm = 760mmHg), 21% *p*O_2_ (with 95% air/5% CO_2_; hyperoxia for term placenta, 160mmHg) or 1% *p*O_2_ (with 5% CO_2_/balance N_2_; hypoxia for term placenta, 7.6mmHg) for 6 days. Culture medium was replaced daily and fresh medium was pre-equilibrated (24h in advance) at each *p*O_2_ before addition to explants. On days 3–5, explants were untreated (control) or treated daily with *p*O_2_-sensitive K^+^ channel blockers 5mM 4-AP or 5mM TEA (these concentrations have been previously reported to produce the maximal inhibitory effect on hCG secretion without effecting tissue integrity [22]), or H_2_O_2_ (10, 100μM or 1mM).

Explant culture medium was collected daily and stored at -20°C before measuring hCG secretion and lactate dehydrogenase (LDH; released from necrotic cells and used as marker of cellular viability).

On day 6 explants were dissolved in 0.3M NaOH at 37°C for 24h to measure protein content. Otherwise explants were placed into water for 18h at room temperature to lyse for measurement of cellular hCG/LDH. The supernatant was collected and stored at -20°C, and explants were dissolved into 0.3M NaOH. These samples were used to measure protein content with Bio-Rad Protein Assay (Bio-Rad Laboratories, Hempstead, UK).

### Measurement of hCG and LDH

hCG was assayed in the explant-conditioned culture medium and in villous explants lysed in water at day 6 of culture using an ELISA (DRG Diagnostics, Marburg, Germany) following the instructions of the manufacturer. hCG secretion was expressed as mIU/ml/h/mg protein.

LDH release into explant-conditioned culture medium culture medium was measured using a cytotoxicity detection kit (Roche Diagnostics, Mannheim, Germany) according to the instructions of the manufacturer. A standard curve was generated using L-Lactic dehydrogenase from rabbit muscle as an internal control. LDH release was expressed as absorbance units/mg protein/h.

### ^86^Rb efflux from placental villous explants

^86^Rb, a tracer of K^+^, permeates most K^+^-selective channels. It has been used to indirectly assess K^+^ permeability of the syncytiotrophoblast [31, 32]. ^86^Rb efflux was measured in placental villous explants using a technique previously described [31]. In principle, the tissue is incubated with ^86^Rb to achieve a stable intracellular level of isotope and then the extracellular ^86^Rb is removed by washing. Efflux of ^86^Rb into ^86^Rb-free buffer is measured over time and expressed either as a proportion of ^86^Rb in the tissue (%^86^Rb efflux) or as the fall in intracellular ^86^Rb (^86^Rb efflux rate constant). Specifically, fragments were incubated for 2h at 37°C in 1ml Tyrode’s buffer (135mM NaCl, 5mM KCl, 1.8mM CaCl_2_, 1mM MgCl_2_, 10mM HEPES, 5.6mM glucose, pH 7.4; osmolality 300mOsm/kgH_2_O) containing 4μCi/ml ^86^Rb (89.7μM; PerkinElmer, Waltham, MA, USA). After incubation, fragments were washed in 15ml Tyrode’s buffer twice for 5min each. Basal ^86^Rb efflux was then measured by changing and collecting 4ml Tyrode’s buffer every 2min for 10min at 37°C. Finally, villi were lysed in water for 18h to release intracellular non-membrane bound ^86^Rb which was then measured in the supernatant to give a measure of total ^86^Rb remaining in the tissue at the end of the experiment (^86^Rb in tissue). Effluxed and tissue ^86^Rb was measured in a beta-counter (Packard 2000, CA, USA).

The time course of percentage (%) ^86^Rb efflux was calculated as:

$$\%\frac{efflux}{2min}=\left(\frac{86Rb\;effluxed}{86Rb\;in\;tissue}\right)\times 100$$

The efflux rate constant was also determined, making the assumption that, in control untreated explants, ^86^Rb efflux at steady state reflects the loss of ^86^Rb from a single compartment (syncytiotrophoblast) limited by the K^+^ permeability of the microvillous membrane. Consequently, the loss of ^86^Rb was measured by a first-order rate constant which was calculated over 16min experimental period as:

$$l_{n}\left(\frac{86Rb\;in\;tissue\;at\;time\;t}{86Rb\;in\;tissue\;at\;start}\right)$$

### Expression of Results and Statistics

Statistical analysis was performed using GraphPad Prism version 5 software. hCG secretion and LDH release from control untreated explants were expressed as mean ± SE (n = number of placentas). Due to variability in hCG secretion between placentas [33], hCG secretion in treated explants at days 4, 5 and 6 of culture was expressed as a percentage of control (established as a 100%) and analysed with a Wilcoxon signed-rank test. A *p* value less than 0.05 was considered statistically significant. Data are median ± interquartile range (IQR).

%^86^Rb efflux from placental villous explants was expressed as mean ± SE for each time point. For all ^86^Rb efflux experiments, significant differences between ^86^Rb rate constants were assessed using least squares linear regression. A *p* value less than 0.05 was considered statistically significant.

## Results

### Effect of *p*O_2_ on hCG secretion from placental villous explants

**Fig 1:**
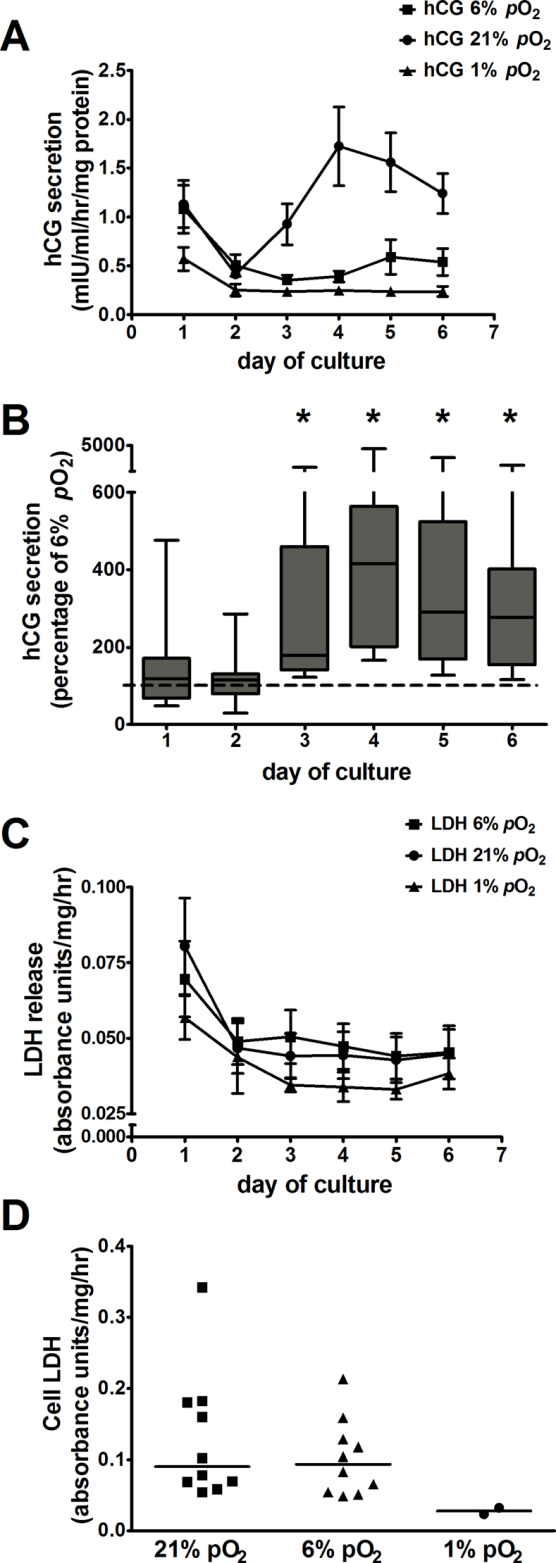
Effect of *p*O_2_ on hCG secretion from villous explants. ***A***: time course of hCG secretion from explants maintained at 21%, 6% and 1% *p*O_2_ during 6 days of culture. Values are mean ± SE; n = 14 placentas (n = 3 placentas maintained at 1% *p*O_2_). ***B***: hCG secretion in explants maintained at 21% *p*O_2_ expressed as a percentage of hCG secretion at 6% *p*O_2_ (100%, dotted line); data are expressed as median ± IQR; n = 14 placentas, Wilcoxon signed-rank test compared to 100%, *p<0.001. ***C***: time course of LDH release from explants maintained at 6%, 21% and 1% *p*O_2_ during 6 days of culture. Values are mean ± SE; n = 14 placentas (n = 3 placentas maintained at 1% *p*O_2_). ***D***: cellular LDH measured at day 6 of culture in explants maintained at 6%, 21% and 1% *p*O_2_. Scatter dot plot shows line at median; n = 10 placentas (n = 2 placentas maintained at 1% *p*O_2_).

The temporal changes in hCG secretion from term placental villous explants maintained at 21% *p*O_2_ over a 6-day culture period (Fig 1*A*) were similar to those previously reported [22, 31].

At all *p*O_2_, hCG secretion was high at day 1 and fell markedly at day 2. Afterwards, hCG secretion increased 4-fold by day 4 in 21% *p*O_2_, showed a slight gradual increase towards the end of culture in 6% *p*O_2_ but remained stable at low values at 1% *p*O_2_ (Fig 1*A*). Compared to hCG secretion at 6% *p*O_2_ (considered to be placental normoxia), secretion was significantly higher (4.1-fold) at 21% *p*O_2_ (Fig 1*B*) but not different at 1% *p*O_2_ (data not shown).

Fig 1*C* shows that after the first day in culture, LDH release declined in explants maintained at 6%, 21% and 1% *p*O_2_, indicating that tissue viability and cellular integrity was maintained in all *p*O_2_. However, the low LDH release at 1% *p*O_2_ might be due to reduced production of the enzyme in hypoxia as cellular LDH was ~3 times lower in 1% than either 21 or 6% *p*O_2_ (Fig 1*D*).

### Effect of *p*O_2_-sensitive K^+^ channel blockers on hCG secretion from placental villous explants

**Fig 2:**
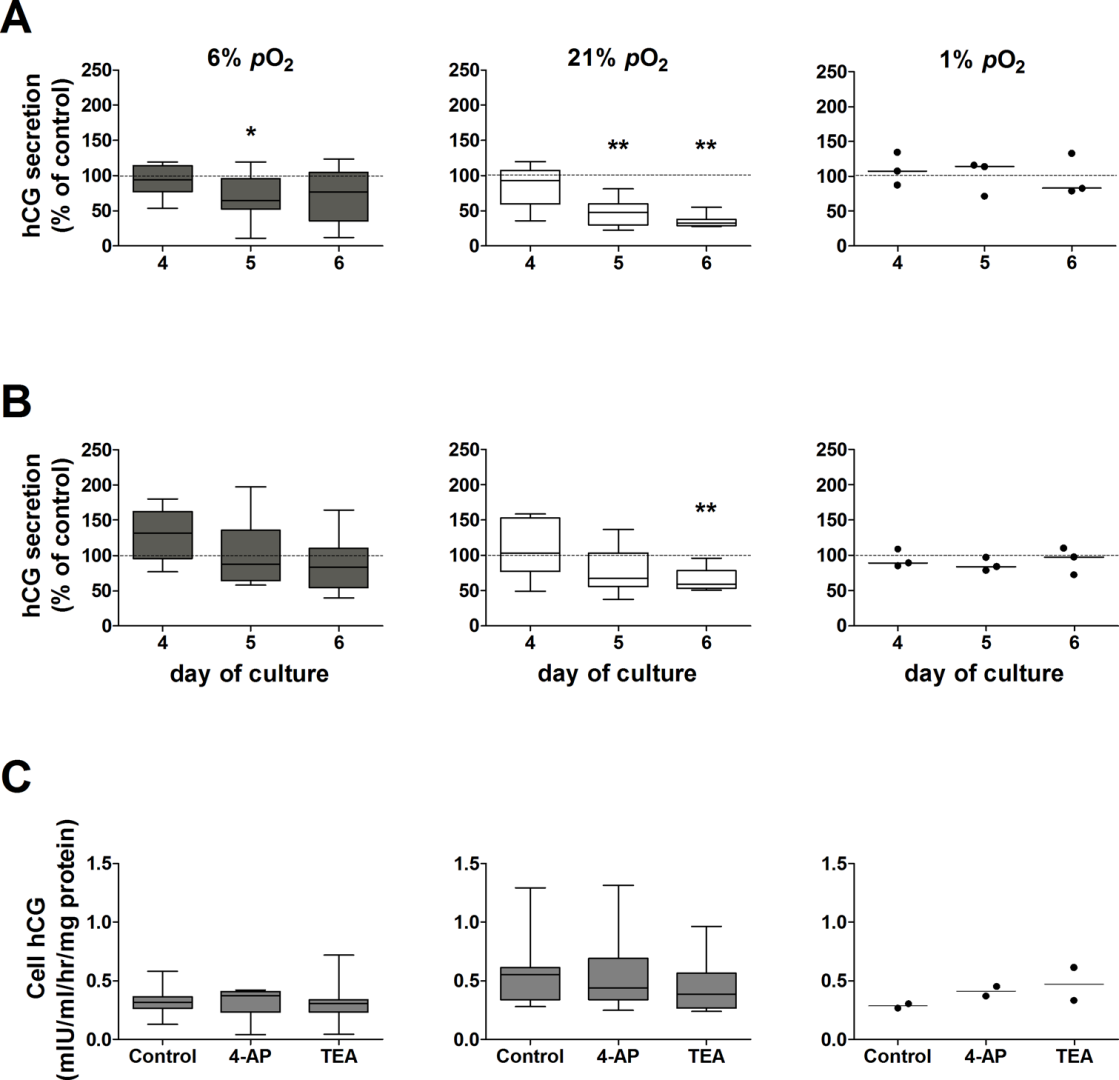
Effect of *p*O_2_-sensitive K^+^ channel blockers on hCG secretion from placental villous explants maintained at 6%, 21% or 1% *p*O_2_. hCG secretion at days 4, 5 and 6 of culture was normalized as a percentage of hCG secretion in control untreated explants at the corresponding *p*O_2_ (dotted line, 100%); assessed by Wilcoxon signed-rank test compared to 100%. ***A***: 4-AP (*p = 0.04, ** *p* = 0.008; n = 8 placentas; 1% *p*O_2_ n = 3 placentas); ***B***: TEA (**p = 0.008, n = 8 placentas; 1% *p*O_2_ n = 3 placentas). Data are expressed as median ± IQR (line at median in 1% *p*O_2_). ***C***: Effect of *p*O_2_-sensitive K^+^ channel blockers on cellular hCG from placental villous explants maintained at 6, 21% or 1% *p*O_2_. Cell hCG was measured at day 6 of culture. Median ± IQR for 6% and 21% *p*O_2_; n = 7 placentas. At 1% *p*O_2_ line represents median; n = 2 placentas.

Fig 2*A* and 2*B* show the effects of *p*O_2_-sensitive K^+^ channel blockers 4-AP and TEA respectively on hCG secretion from placental villous explants maintained at 6%, 21% and 1% *p*O_2_.

In villous explants maintained at 6% *p*O_2_, 4-AP caused a transient decrease (35%) in hCG secretion on day 5 compared to control untreated explants (Fig 2*A*). In contrast, explants maintained at hyperoxia (21% *p*O_2_) showed a significant reduction in hCG secretion when treated with 4-AP at days 5 (52%) and 6 (68%) of culture (Fig 2*A*). This effect was completely suppressed under hypoxia (1% *p*O_2_), where hCG secretion was unaffected by 4-AP (Fig 2*A*).

TEA did not affect hCG secretion by explants maintained in placental normoxia (Fig 2*B*). On the contrary, when explants were maintained at 21% *p*O_2_, treatment with TEA caused a significant reduction in hCG secretion at day 6 of culture (41%; Fig 2*B*). TEA had no effect on explants maintained at 1% *p*O_2_ (Fig 2*B*).

LDH release from placental villous explants was not affected by treatment with 4-AP or TEA compared to their corresponding controls at the same *p*O_2_ (data not shown), indicating that tissue viability was not compromised by treatment with these *p*O_2_-sensitive K^+^ channel blockers.

Neither 4-AP nor TEA altered cellular hCG at any of the *p*O_2_ tested (Fig 2*C*), implicating an effect of these K^+^ channel blockers on the secretory mechanism for hCG and not hCG production.

From these data it is evident that culture of term placental villous explants for 6 days in hypoxic (1% *p*O_2_) conditions reduced hCG secretion to a low level, inhibited the temporal recovery in hCG secretion which is associated with syncytiotrophoblast regeneration/renewal at higher *p*O_2_ and reduced the cellular production of LDH and hCG. Furthermore, hCG secretion at 1% *p*O_2_ was unaffected by 4-AP and TEA. Therefore, experiments to evaluate the effect of these K^+^ channel blockers on ^86^Rb efflux were not performed at 1% *p*O_2_.

### Basal ^86^Rb (K^+^) efflux from syncytiotrophoblast: chronic effect of *p*O_2_

**Fig 3:**
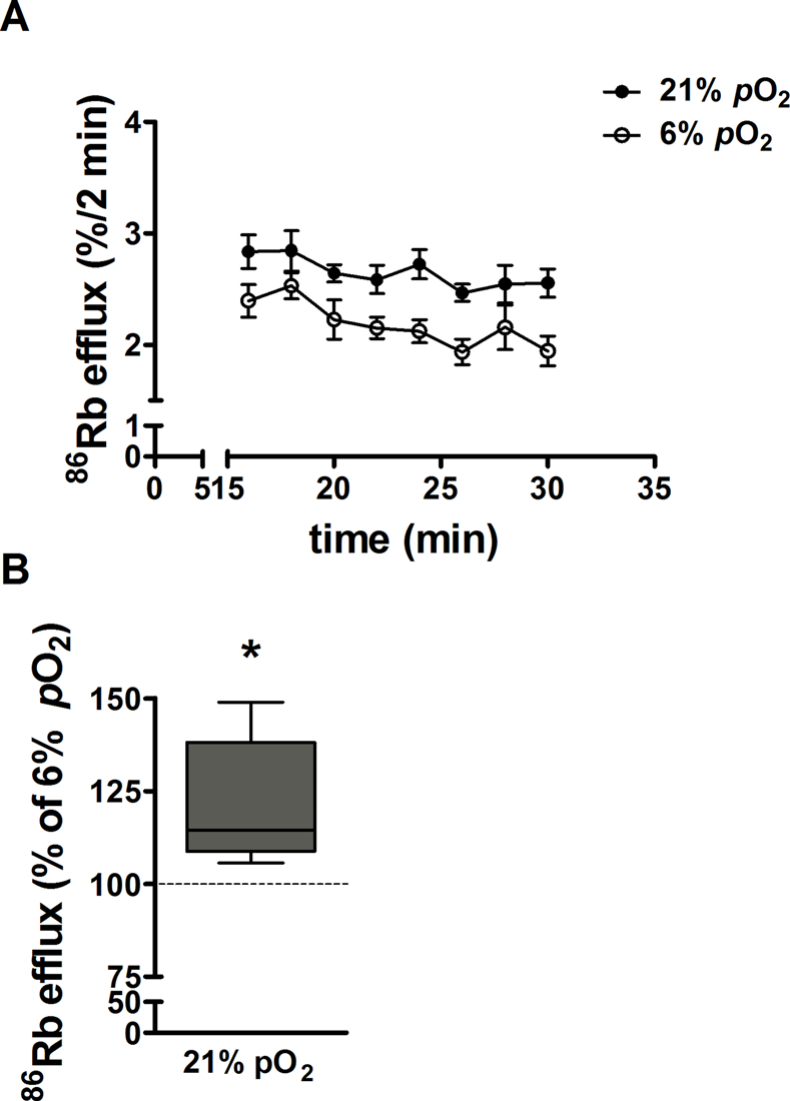
Effect of *p*O_2_ on syncytiotrophoblast K^+^ permeability. ***A***: Time course for the %^86^Rb efflux over 16min. Untreated (control) villous explants were cultured at 6% and 21% *p*O_2_ and basal ^86^Rb efflux was measured at day 6. Data are expressed as mean ± SE (n = 10 placentas). ***B***: The %^86^Rb efflux over 16min in explants maintained at 21% was expressed as a percentage of efflux in explants maintained at 6% *p*O_2_ (placental normoxia; dotted line). Data are median ± IQR, Wilcoxon signed-rank test compared to 6% *p*O_2_, *p = 0.002; n = 10 placentas.

^86^Rb efflux was measured at day 6 of culture in placental villous explants maintained at 6% or 21% *p*O_2_. Fig 3*A* shows the time course for basal %^86^Rb efflux at a steady state over a 16min period in explants maintained at 6% and 21% *p*O_2_. %^86^Rb efflux is higher from explants maintained at 21% than 6% *p*O_2_. Fig 3*B* shows the area under the curve for the total %^86^Rb efflux over 16min from explants maintained at 21% *p*O_2_ as a percent of efflux from explants at 6% *p*O_2_ (100%, dotted line). Basal %^86^Rb efflux was significantly higher in explants maintained for 6 days in hyperoxia (21% *p*O_2_) compared to normoxia.

The ^86^Rb efflux rate constants calculated for untreated control explants maintained at 6% and 21% *p*O_2_ is shown in Table 1. Rate constant analysis shows that the fall in intracellular ^86^Rb can be described by a single exponential decline indicating that efflux is predominantly from a single tissue compartment, which we take to be the syncytiotrophoblast, in agreement with previous reports [31]. The mean rate constant for ^86^Rb efflux was significantly lower in explants maintained at placental normoxia (6% *p*O_2_) than hyperoxia (21% *p*O_2_) (Table 1).

**Table 1:** Rate constants of ^86^Rb efflux in control and treated placental villous explants.

*Condition*	^*86*^*Rb efflux rate constant(ln* ^*86*^*Rb (t = x)/(t = 0)/min*^*-1*^*)*	*r*^*2*^	*p value*	*n*
Control 6% *p*O_2_	-0.0111 ± 0.0003	0.956		10
Control 21% *p*O_2_	-0.0136 ± 0.0003*	0.954	<0.0001	10
Control for treatments 6% *p*O_2_	-0.0107 ± 0.0005	0.948		4
5mM 4-AP 6% *p*O_2_	-0.0114 ± 0.0009†	0.840	0.494	4
5mM TEA 6% *p*O_2_	-0.0111 ± 0.0005†	0.944	0.630	4
100μM H_2_O_2_ 6% *p*O_2_	-0.0119 ± 0.0003†	0.976	0.048	4
1mM H_2_O_2_ 6% *p*O_2_	-0.0118 ± 0.0007†	0.916	0.002	4
Control for treatments 21% *p*O_2_	-0.0144 ± 0.0006	0.953		4
5mM 4-AP 21% *p*O_2_	-0.0113 ± 0.0006**	0.918	0.0007	4
5mM TEA 21% *p*O_2_	-0.0117 ± 0.0002**	0.987	<0.0001	4
100μM H_2_O_2_ 21% *p*O_2_	-0.0122 ± 0.0004**	0.963	0.004	4
1mM H_2_O_2_ 21% *p*O_2_	-0.0130 ± 0.0005**	0.963	0.014	4

**Rate constants of** ^**86**^**Rb efflux in control and treated placental villous explants maintained at 6% and 21% *p*O**_**2**_ **over 16 min.** Data are mean ± SE; n is the number of placentas. In all conditions the r^2^ values, determined by linear regression, were close to 1 and significant (*p*<0.001), indicating a single exponential decline in intracellular ^86^Rb over 16 min. The *p* value corresponds to the following differences in the rate constants between groups: *control 6% *p*O_2_ vs control 21% *p*O_2_; †treatments at 6% *p*O_2_ vs corresponding control at 6% *p*O_2_; **treatments at 21% *p*O_2_ vs corresponding control at 21% *p*O_2_.

### Long term effects of *p*O_2_: effect of *p*O_2_-sensitive K^+^ channel blockers on syncytiotrophoblast K^+^ permeability

^86^Rb (K^+^) permeability was assayed at day 6 in villous explants cultured at 6% and 21% *p*O_2_. Explants were untreated (controls) or treated from day 3 onwards with 4-AP or TEA.

The effect of the K^+^ channel blockers was assessed by analysing the differences between the rate constant of decline in intracellular ^86^Rb for each treatment compared to controls (performed in the same placentas n = 4) at the same *p*O_2_ (Table 1). The efflux rate constant was significantly reduced by 4-AP and TEA in explants maintained in hyperoxia (21% *p*O_2_) but was without effect in explants maintained in normoxia (6% *p*O_2_) (Table 1).

### Effect of H_2_O_2_ on basal ^86^Rb (K^+^) permeability and hCG secretion from placental villous explants

**Fig 4:**
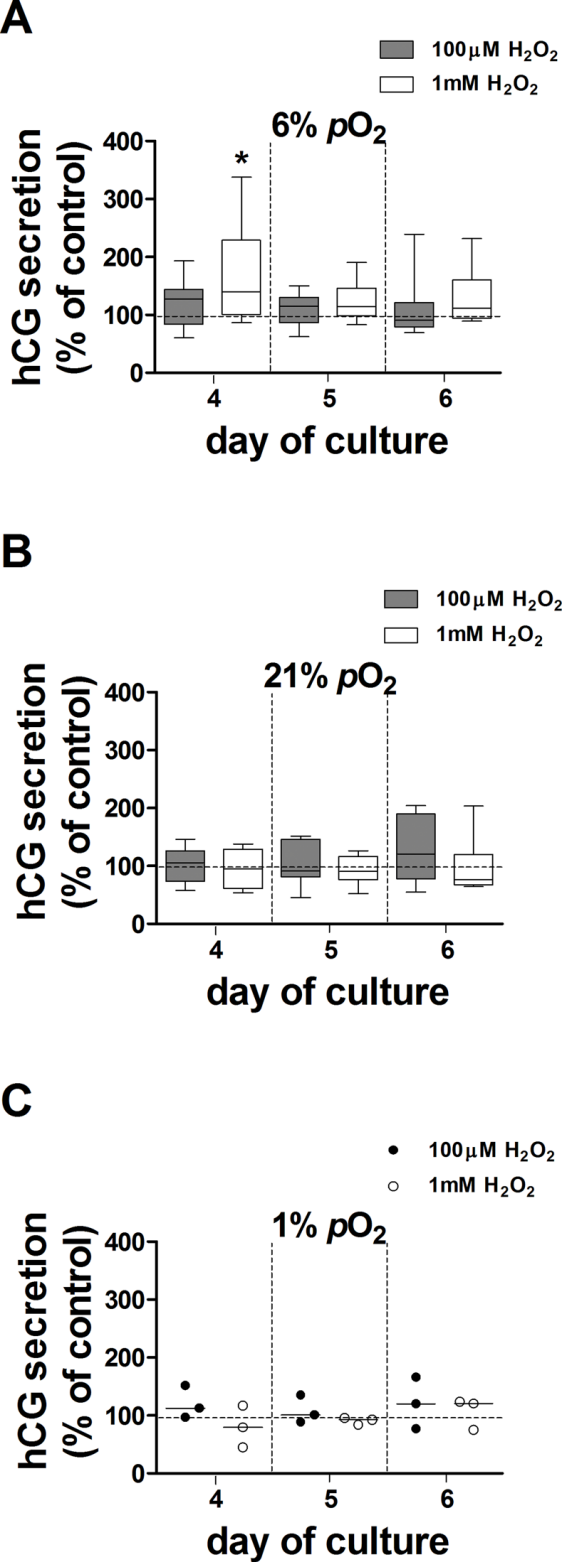
Effect of 100μM and 1mM H_2_O_2_ on hCG secretion from villous explants. hCG secretion from H_2_O_2_-treated explants maintained at 6% (***A***), 21% (***B***) and 1% *p*O_2_ (***C***) at days 4, 5 and 6 of culture was expressed as a percentage of control (100%, dotted line); data are expressed as median ± IQR; n = 9 placentas for 100μM H_2_O_2_ (except n = 3 placentas at 1% *p*O_2_); n = 8 placentas for 1mM H_2_O_2_ (except n = 3 placentas in 1% *p*O_2_). Wilcoxon signed-rank test compared to 100%, *p = 0.04.

The effect of H_2_O_2,_ used to generate oxidative stress, on hCG secretion was tested in explants maintained at 6%, 21% and 1% *p*O_2_ over days 3–5 of culture. There was no effect of 100μM H_2_O_2_ at 6%, 21% or 1% *p*O_2_ (Fig 4). In contrast, 1mM H_2_O_2_ transiently increased hCG secretion by 40% in explants maintained at 6% *p*O_2_ compared to controls (Fig 4*A*). 10μM H_2_O_2_ had no effect on hCG secretion (data not shown). Treatment with H_2_O_2_ did not affect LDH release from villous explants at any of the concentrations used (data not shown).

The effect of H_2_O_2_ on ^86^Rb efflux was measured at day 6 in explant cultures maintained at 6% and 21% *p*O_2_ (Table 1). H_2_O_2_ increased the ^86^Rb efflux rate constant at 100μM and 1mM compared to corresponding controls in explants maintained at 6% *p*O_2_. In contrast, treatment of villous explants with 100μM and 1mM H_2_O_2_ produced the opposite effect in 21% *p*O_2_, and significantly reduced the ^86^Rb rate constant (Table 1) compared to controls at the same *p*O_2_. 10μM H_2_O_2_ had no effect on basal ^86^Rb efflux from explants cultured at either 6% or 21% *p*O_2_ (data not shown).

## Discussion

This study confirms and extends previous observations that hCG secretion from term placental trophoblast is sensitive to *p*O_2_ [8, 18] and ROS [10]. In villous explants prepared from the same placenta, hCG secretion was higher in 21% *p*O_2_, and lower in 1% *p*O_2_, than 6% *p*O_2_ (normoxic) culture conditions. Syncytiotrophoblast K^+^ permeability, estimated by ^86^Rb efflux, was higher in explants cultured in 21% than 6% *p*O_2_. In accordance with this, 4-AP and TEA, blockers of *p*O_2_-sensitive K_V_ channels, inhibited hCG secretion and ^86^Rb efflux to a greater extent in 21% than 6% *p*O_2_. H_2_O_2_, used to induce oxidative stress, stimulated ^86^Rb efflux and transiently increased hCG secretion from explants cultured at 6% *p*O_2_ but inhibited ^86^Rb efflux, without affecting hCG secretion, at 21% *p*O_2_. Thus, effects of oxidative stress on hCG secretion and syncytiotrophoblast K^+^ permeability depend on the *p*O_2_.

### hCG secretion from term placental syncytiotrophoblast is *p*O_2_-dependent

The temporal pattern of hCG secretion from term placental explants maintained at 21% *p*O_2_ for 6 days was originally described by Siman *et al*. [31]. Similar studies to relate hCG secretion to syncytiotrophoblast regeneration over 6 days at placental normoxia (6% *p*O_2_), and extreme hypoxia (1% *p*O_2_), have not been performed. However, in shorter term cultures (4 days), hCG secretion was lower at 6% and 1% compared to 21% *p*O_2_ and this was associated with dysregulated syncytiotrophoblast turnover such as decreased cytotrophoblast proliferation and enhanced apoptosis [18]. In the present study, the time course of hCG secretion from explants maintained at 21% *p*O_2_ coincided with previous observations [22, 31]. Secretion was significantly lower at 6% *p*O_2_, with only a small rise on day 4, and at 1% *p*O_2_ low hCG secretion at day 2 persisted for the duration of the culture. Cellular hCG was lower (1.7-fold) in explants maintained at 6% *p*O_2_ compared to 21% *p*O_2_, suggesting that *p*O_2_ regulates hCG synthesis. However, as hCG secretion at 6% *p*O_2_ was 4.1-fold lower than at 21% *p*O_2_, the hCG secretory mechanism is additionally regulated by *p*O_2_. In contrast, 1% *p*O_2_ reduced cellular hCG to the same proportion as secretion, indicating that the reduced secretion in hypoxia is predominantly due to altered synthesis. LDH release was unaffected by *p*O_2_ which could indicate that extremes of *p*O_2_ do not markedly alter tissue integrity. However, culturing villous tissue at 1% *p*O_2_ inhibited LDH synthesis and using LDH release alone as a marker of tissue viability in hypoxia might not be reliable.

### Inhibition of hCG secretion by K_V_ channel blockers is *p*O_2_-sensitive

We previously demonstrated that chronic exposure to the K_V_ channel blockers 4-AP and TEA induced a concentration-dependent inhibition of hCG secretion in explants and cytotrophoblasts maintained at 21% *p*O_2_ [22]. In the current study, 4-AP and TEA inhibited hCG secretion from villous explants to a greater extent in 21% than 6% *p*O_2_ and had no effect on secretion at 1% *p*O_2_. As the activity and expression of K_V_ channels can be down-regulated by hypoxia [24, 25], our results support the possibility that the lower hCG secretion at 6% compared to 21% *p*O_2_ is mediated by closure of K_V_ channels.

### Inhibition of ^86^Rb (K^+^) efflux by K_V_ channel blockers is *p*O_2_-sensitive

Direct study of ion channels in the syncytiotrophoblast of intact placental villi using patch clamp methods is technically challenging as seals are hard to achieve [34] and the multinucleate nature of the tissue precludes whole cell recording. In this study we used ^86^Rb (K^+^) efflux to assess whether 4-AP and TEA inhibited K^+^ conductance in the syncytiotrophoblast and whether the inhibition was *p*O_2_-sensitive. We have previously shown that basal ^86^Rb efflux from placental explants cultured at 21% *p*O_2_ is inhibited by Ba^2+^, a broad spectrum K^+^ channel blocker [31], implicating K^+^ conductances in the microvillous, maternal facing plasma membrane of the syncytiotrophoblast.

In the current study, ^86^Rb efflux measured on day 6 of culture showed that basal K^+^ permeability was significantly higher in explants maintained in hyperoxia compared to placental normoxia, suggesting that chronic exposure to 21% *p*O_2_ over a 6-day period increases the activity and/or expression of syncytiotrophoblast K^+^ channels. In support of this, treatment of villous explants with 4-AP and TEA significantly reduced ^86^Rb efflux when the tissue was cultured at 21% but not 6% *p*O_2_, consistent with an inhibition of *p*O_2_-sensitive K_V_ channels that are more active/more highly expressed at 21% than at 6% *p*O_2_. In addition, the inhibition of both ^86^Rb efflux and syncytiotrophoblast hCG secretion by 4-AP and TEA at 21% but not 6% *p*O_2_, suggests that 4-AP and TEA-sensitive K_V_ channels mediate the stimulatory effect of higher *p*O_2_ on hCG secretion.

### Effect of H_2_O_2_ on hCG secretion and ^86^Rb efflux

Placental oxidative stress and reduced antioxidant defences are key features of pre-eclampsia [35, 36]. In this study we explored the effects of oxidative stress (H_2_O_2_) on syncytiotrophoblast hCG secretion and whether they could be modulated through K^+^ channels. Previous reports showed that *in vitro* treatment of placental villous tissue with 1mM H_2_O_2_ caused oxidative stress which was reversed by vitamins C and E [37].

H_2_O_2_ (10μM-1mM) did not alter hCG secretion at 21% *p*O_2_ but 1mM caused a transient increase at 6% *p*O_2_. This contrasts with the concentrations of H_2_O_2_ reported to affect hCG secretion by cytotrophoblasts (inhibition at >50μM and stimulation at 1–50μM) perhaps due to differences between these *in vitro* preparations; in explants cellular interactions are maintained and tissue antioxidant defences are available to scavenge ROS [38].

H_2_O_2_ also had *p*O_2_-dependent effects on ^86^Rb efflux, with 100μM-1mM increasing syncytiotrophoblast ^86^Rb efflux at 6% *p*O_2_, but inhibiting efflux at 21% *p*O_2_. This is consistent with the variable effects of ROS on K^+^ channel activity reported on the literature [28, 30] and raises the possibility that in placental normoxia, K^+^ channels can be activated by H_2_O_2_. However, it is possible that the effect of H_2_O_2_ on ^86^Rb efflux and hCG secretion can be independent events and further work is required to determine which channels are activated by H_2_O_2_ in normoxia and whether they are involved in hCG secretion_._

### Mechanism of hCG secretion: Role of K^+^ channels

In contrast to hCG secretion in the first trimester of pregnancy [19], the mechanism of secretion by the syncytiotrophoblast at term is not fully elucidated. The present work proposes a role for 4-AP and TEA-sensitive *p*O_2_-sensitive K^+^ channels in regulating hCG secretion. According to the specificity and the concentration of 4-AP and TEA used, the targeted K^+^ channels belong mainly to the K_V_ channel family [23]. Indeed, K^+^ channels belonging to other families such as ATP-sensitive K^+^ channels are not involved in hCG secretion [39].

K_V_ channel mRNA is expressed by whole placental homogenate [40, 41] and immunostaining for K_V_1.5 and 2.1 has localized protein expression to the syncytiotrophoblast [42]. K_V_1.5 and 2.1 are *p*O_2_-sensitive [43] and blocked by 4-AP and TEA [44] and thus closure of these channels could underlie the lower hCG secretion from placentas maintained in 6% compared to 21% *p*O_2_.

In the normal placenta at term where villi are exposed to maternal blood at 6% *p*O_2_, *p*O_2_-sensitive K_V_ channels could be down-regulated/closed. We have previously shown that hCG secretion at 21% *p*O_2_ is stimulated by Ca^2+^ entry through non-selective cation channels (NSCC; [45]). Therefore, a relatively depolarised membrane potential could minimise Ca^2+^ entry through NSCC and sustain basal levels of syncytiotrophoblast hCG secretion seen under normoxic conditions. Elevated K^+^ permeability by ROS (H_2_O_2_) in normoxia could reflect increased K^+^ channel activity, membrane hyperpolarization, promotion of Ca^2+^ entry and stimulation of hCG secretion.

In conditions such as pre-eclampsia, a pregnancy complication associated with altered *p*O_2_ [13], whilst the range of *p*O_2_ in the placental bed is unlikely to be as wide as that used *in vitro* in this study, current data suggest the syncytiotrophoblast could be exposed to both hypoxia [46] and/or hyperoxia [47, 48]. In the latter, *p*O_2_-sensitive K_V_ channels could be activated, hyperpolarising the membrane potential, stimulating Ca^2+^ entry and promoting syncytiotrophoblast hCG secretion. In this regard it is interesting to note that maternal plasma hCG is higher in women with late onset pre-eclampsia compared to women having normal pregnancy [16], and that serum hCG levels vary depending on the severity of disease showing a several-fold increase in severe [49] but not in moderate pre-eclampsia. Consequently, *p*O_2_ changes in the placenta might differ related to the severity of disease and this could influence the regulation of hCG secretion by elevated ROS.

Several mechanistic links remain to be explored. For example, although hypoxia inhibits cytotrophoblast cell fusion and hCG secretion [9], there is insufficient evidence at present to confirm that these events are independently *p*O_2_-sensitive. Using primary cultures of placental trophoblast *in vitro*, Alsat *et al*. (1996) demonstrated that low oxygen (~9% *p*O_2_) reduced the formation of multinucleate cells (syncytialisation) and this was associated with an increase in expression of desmoplakin and e-cadherin, and a reduction in hCG secretion. While these data illustrate a clear effect of oxygenation on morphological and biochemical differentiation of cytotrophoblasts, it is unclear whether the primary effect is to reduce hCG secretion, which then inhibits fusion, or whether the primary effect of low *p*O_2_ is to inhibit fusion which prevents biochemical differentiation. Furthermore, it is also unknown whether the expression/activity of *p*O_2_-sensitive K_V_ channels is altered either by cytotrophoblast differentiation, or *p*O_2_, per se.

The extent to which *p*O_2_-sensitive K^+^ channels play a role in hCG secretion in pregnancy disease has yet to be explored; dysregulation of syncytiotrophoblast K^+^ channel activity and/or expression through chronic exposure to altered *p*O_2_ and/or increased ROS could potentially contribute to altered trophoblast renewal and hCG secretion in pre-eclampsia.
